# Early postnatal vocalizations predict sociability and spatial memory in C57BL/6J mice: Individual differences in behavioral traits emerge early in development

**DOI:** 10.1371/journal.pone.0186798

**Published:** 2017-11-01

**Authors:** Kaichi Yoshizaki, Kohei Koike, Ryuichi Kimura, Noriko Osumi

**Affiliations:** 1 Department of Developmental Neuroscience, United Centers for Advanced Research and Translational Medicine, Tohoku University Graduate School of Medicine, Sendai, Japan; 2 Department of Pathology, Institute for Developmental Research, Aichi Human Service Center, Aichi, Japan; 3 Department of Physiology, Center for Integrative Physiology and Molecular Medicine, Saarland University School of Medicine, Homburg, Germany; Tokyo Metropolitan Institute of Medical Science, JAPAN

## Abstract

The understanding of individual diversity and its link to brain functions is a fundamental issue in neurobiology. Studies in mice have mainly focused on the investigation of behavior traits in adulthood, whereas longitudinal analyses are largely uninvestigated. Here we have conducted systematic behavior tests in individual mice (C57BL6/J, male), comparing phenotypes at early postnatal stages and in adulthood. Each animal showed different scores in individual behavior tests. However, we observed an inverse correlation between repetitive behavior in the Morris water maze test and sociability in the 3-chamber social interaction test; an increase in repetitive behaviors was associated with poor sociability. In longitudinal analyses, the emission of ultrasonic vocalization during maternal separation at postnatal day 6 in pups was correlated positively with sociability and negatively with spatial memory. Our results show a possibility that individual differences in communication between pups and their mother in infancy is a predictive indicator for sociability and cognitive performance as an adult.

## Introduction

How personality develops over a lifetime and how it interacts with specific cognitive traits in adulthood have been important issues in psychology, which have now come into focus in neuroscience. The nature of personality is generally defined on the basis of certain observable behavior characteristics [1]. To date, issues in development of personality have been addressed mainly in human studies, by observing identical twins, to understand the additive or interacting contributions of genetic and environmental variation to individual differences in behavioral development [2, 3]. Recently, cognitive traits of inbred animal models have been used to understand “personality” at the biological level [4]. For instance, a specific link has been revealed between working memory and sensorimotor gating in inbred C57BL/6NCrl mice [5], which has not been reproduced in another study [6]. These rodent studies demonstrate that some behavioral traits are significantly associated in adulthood. However, such individual traits in adulthood have not been traced back to early childhood.

In early childhood, behavioral tests in pups are limited due to their immature motor functions. However, researchers have recently focused on ultrasonic vocalization (USV). Rats and mice at the suckling stage elicit USV when they are separated from their dam and littermates. This behavioral trait is currently used to examine the early communicative behavior of the pup-mother dyad and as a sign of an aversive affective state [7]. Many neurotransmitters and neuropeptides are involved in the regulation of USV in rodents, including serotonin (5-HT) [8], cannabinoids (CBs) [9], GABA [10, 11], oxytocin [12] and vasopressin [13]. Furthermore, an altered USV emission rate and a delayed ontogenetic profile of USV emission were reported in models of neurodevelopmental diseases, including autism spectrum disorder (ASD) [14–18]. Thus, maternal separation-induced USV was assumed to reflect functions of various brain regions and used as a useful tool to examine individual traits in early childhood.

In the present study, we performed systematic behavioral analyses in inbred C57BL6/J male mice at the early postnatal stage and in adulthood. We have demonstrated an important association between repetitive behavior and sociability and cognition in adulthood. Surprisingly, the frequencies of USV calls at the early postnatal stage were predictive of the sociability and spatial memory in adult mice. This is the first step to understand how individual differences emerge in a genetically homogeneous strain of mice. Further studies using other strains of mice or other species would be necessary to make a robust conclusion.

## Materials and methods

### Animals and ethic statement

All experimental procedures were approved by Ethic Committee for Animal Experiments of Tohoku University Graduate School of Medicine (#2014–112) and animals were treated according to the National Institutes of Health guidance for the care and use of laboratory animals. We purchased 11-week-old male and 9-week-old female inbred C57BL6/J mice from Charles River Laboratories. After one week of habituation, male mice (3-months-old) were mated with virgin female mice (10-weeks-old) for up to one week and separated from the female mice to minimize possible confounding factors against behavior of offspring. The mean litter size was 7.2 ± 1.3 pups, and body weight of the offspring at postnatal day 6 was 3.28 ± 0.41 g. Twenty four offspring male mice were tested with a behavioral test battery. On postnatal day 6, each offspring was tattooed with an Aramis Animal Microtattoo System (Natsume Co., Ltd., Tokyo, Japan) for individual recognition after ultrasonic vocalization recording. All animals were housed in standard cages in a temperature and humidity-controlled room with a 12-hour light/dark cycle (light on at 8:00) and had free access to standard lab chow and tap water.

### Behavioral tests

Ultrasonic vocalization was measured on postnatal day 6 and other behavior tests were conducted at 3-months-old, according to the order described in a previous study, i.e., the prepulse inhibition test, the 3-chamber social interaction test, and then the Morris water maze test [19, 20].

#### Ultrasonic vocalization

Each pup was assessed for ultrasonic vocalization (USV) on postnatal day 6 (P6) according to previously described protocols [7, 21, 22]. The pups were separated from their mother and littermates, one at a time, and placed in a plastic dish with wood chip in a soundproof chamber, and USV calls were recorded for 5 min with a microphone connected with the UltraSound Gate 416H detector set (Avisoft Bioacoustics, Germany) at 25–125 kHz to measure the number of USV calls. The number of USV calls was defined as “vocalization”. USV calls were recorded from all pups born to 5 dams. Only data from male pups were further analyzed in the present study.

#### Prepulse inhibition test

The prepulse inhibition (PPI) test was conducted as described previously [23]. The mice were introduced into a plastic cylinder of a startle chamber (SR-LAB, San Diego Instruments, San Diego, CA). After 5 min acclimatization with 65 dB broadband background noise, PPI test session with a total of 64 trials was conducted. The test session consisted of five types of trials as follows: no stimulus trials (background noise only); startle pulse alone trials (40 ms duration at 120 dB, p120); and three prepulse+pulse trials (20 ms duration prepulse at 68 dB [pp3], 71 dB [pp6] or 77 dB [pp12], followed by a 40 ms duration startle stimulus at 120 dB after a 100 ms delay). Each trial was presented in a pseudo random manner by the SR-LAB software system. The startle response was detected by an accelerometer under the plastic cylinder and recorded from electronic signals.

#### Three-chamber social interaction test

The three-chamber social interaction (3CSI) test was performed according to a previously report [24]. Each chamber was 40 x 60 x 20 cm and divided into three small compartments by a clear Plexiglas wall with small square openings (7 x 7 cm) allowing access into each chamber. An unfamiliar C57BL6/J male (stranger) that had no prior contact with the subject mice was placed in one of the side chambers. The location of stranger in the left or right side chamber was systematically alternated between trials. The stranger mouse was enclosed in a small, round cage which allowed olfactory, visual, auditory, and tactile contacts but did not allow deep contacts. The subject mouse was first placed in the center chamber for 5 min and then allowed to explore the entire social test apparatus without any stranger mouse for 10 min. Then the mice explored the apparatus with stranger mouse in a side chamber. Time spent with stranger mice was defined as “sociability”. Then another stranger mouse was used as a novel mouse and the same mouse that was examined in the previous test was used as a familiar mouse. Time spent with the novel stranger mouse was defined as “social novelty”. Time spent in each chamber was recorded using Any-maze (Brain Science. Idea. Co., Ltd., Japan).

#### Morris water maze test

The Morris water maze (MWM) test was performed as described previously with some modifications [25]. Briefly, circular pool with 1 m diameter was filled with opaque water and the water temperature was maintained at 22°C using a thermostat. The pool was surrounded by curtain screens that had distinctive markings on the surface to enhance spatial location. The pool was monitored by a video camera connected to an auto tracking system (WaterMaze, ActiMetrics, IL). In this study, we trained mice for 9 days to test for spatial learning and reversal learning. First, the mice were trained for 4 days in the pool to locate a transparent circular platform with 10 cm diameter that was submerged 1–1.5 cm beneath the surface of the water. During the training period, the mice experienced two trials of the swimming session per day. On the day 5, the mice freely swam in the pool without the platform for 1 min to test their spatial memory. From day 6, the mice were trained for 3 days in the pool to locate a new position of the platform that was moved to opposite side from the first position. On the day 9, the mice freely swam in the pool without the platform for 1 min to test their ability to reverse a spatial memory once established. Spatial memory was calculated as the proportion of time spent in the original target quadrant in the probe test. Reversal memory and repetitive behavior was calculated as the ratio of time spent in the reversed target to the original target quadrant in the reversal probe test.

### Statistical analyses

All of the available behavioral data except for the USV test and social novelty in 3CSI were reported using the 24 subjects. The sample size for the USV test and social novelty in 3CSI was 23 due to loss of one animal’s data because of a technical failure on the test day. Therefore, we estimated our sample size was enough to detect correlations with 80% power at the 0.05 level of significance. The normality in each behavioral data was derived using the D’Agostino-Pearson test. Pearson correlation analysis was used to determine the relationship to each other, and a *p* value less than 0.05 was considered to be statistically significant.

## Results

### Systematic behavioral analyses using identical animals were conducted at both early postnatal and adulthood

**Fig 1:**
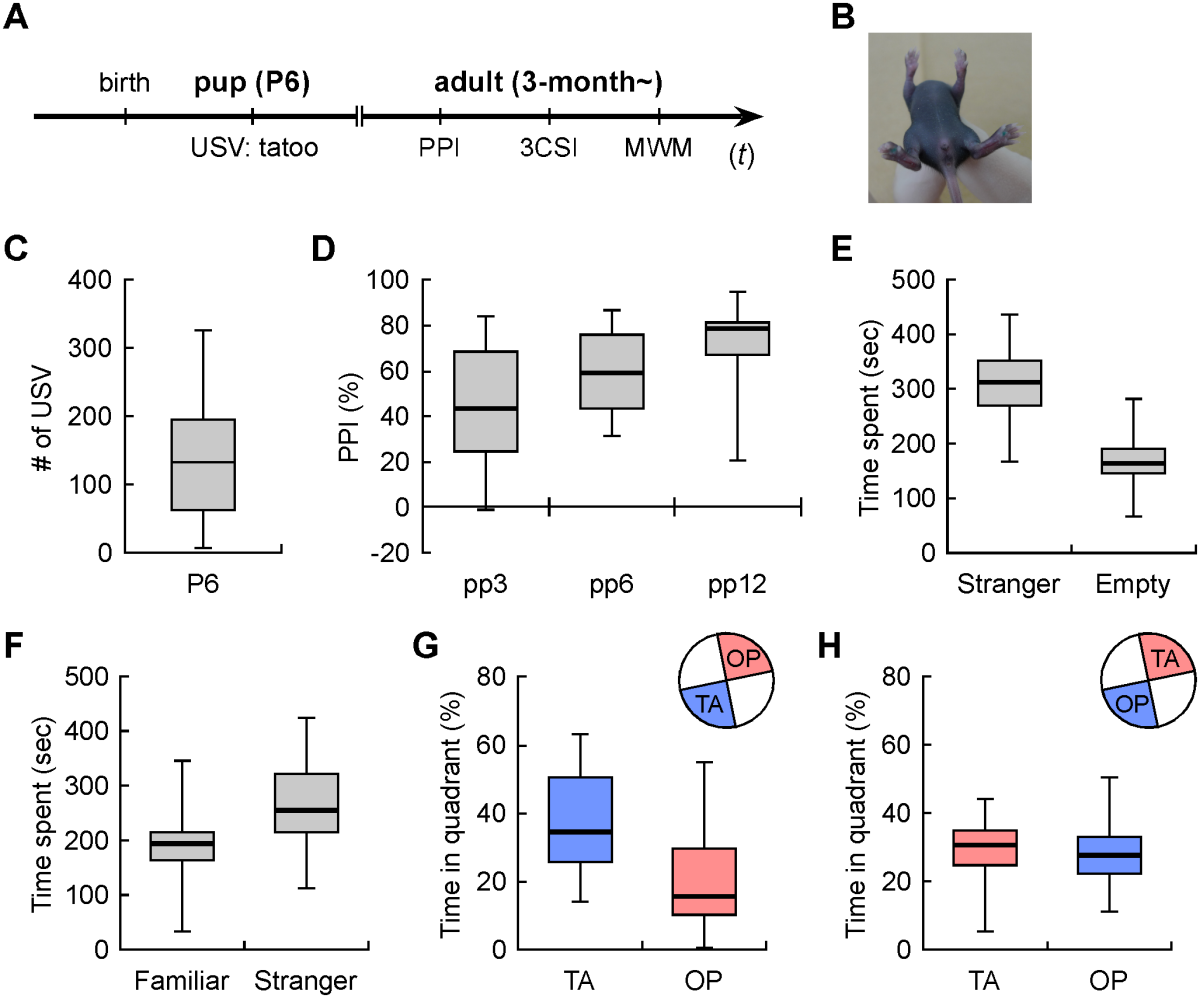
Systematic behavioral analyses using individually identified mice for correlation studies. (A) Experimental design. (B) Representative image of tattoo made on the bottom of hind paws of mouse pups after ultrasonic vocalization (USV) test at P6. (C) Number of USV calls during 5 min after maternal separation. (D) Inhibition of startle response by pulse (120 dB) with weak pre-pulse (68, 71, 77 dB corresponding to pp3, pp6, pp12, respectively). (E) Time spent with stranger mice to the empty cage [sociability] in 3-chamber social interaction test (3CSI). (F) Time spent with stranger mice to familiar mice [social novelty] in 3CSI. (G) Percentage of time in the original (TA) and opposite (OP) target quadrant in probe test in the Morris water maze (MWM). (H) Percentage of time in the reversal (TA) and original (OP) target quadrant in reversal probe test in MWM. All data are presented as boxplots. TA; target area, OP; opposite area.

To examine individual diversity in inbred mice (C57BL6/J, male), we performed a systematic behavioral analysis (Fig 1A) with individual discrimination by combinatorial tattoo on limbs (Fig 1B). In the maternal separation-induced USV test, we counted the number of USV for 5 min after maternal separation (Fig 1C, 142.9 ± 99.7, max: 326, min: 7, normality: χ^2^ = 1.6918, *p* = 0.4292). In the PPI test, we measured the startle response to pulse (120 dB) with a weaker prepulse (68, 71, 77 dB) or no prepulse, and calculated %PPI (Fig 1D, 45.53 ± 24.60, max: 83.99, min: -1.46, normality: χ^2^ = 1.7887, *p* = 0.4089 in 68 dB, 59.30 ± 17.38, max: 87.04, min: 31.52, normality: χ^2^ = 1.7603, *p* = 0.4147 in 71 dB, 73.13 ± 15.51, max: 95.20, min: 20.74, normality: χ^2^ = 15.3087, *p* = 0.0005 in 77 dB). In the 3CSI test, we recorded the time spent with a novel mouse to an empty cage [sociability (Fig 1E, 312.6 ± 63.7, max: 438.0, min: 166.5, normality: χ^2^ = 0.4307, *p* = 0.8063)] and the time spent with a novel stranger mouse to the familiar mouse [social novelty (Fig 1F, 268.3 ± 72.7, max: 409.9, min: 114.0, normality: χ^2^ = 0.0610, *p* = 0.9700)], respectively. In the MWM test, we calculated the percentage of time spent in the original target quadrant in probe test [spatial memory (Fig 1G, 36.94 ± 14.14, max: 63.24, min: 14.33, normality: χ^2^ = 1.6196, *p* = 0.4450)], in the reversed target quadrant in reversal probe test [reversal memory (Fig 1H *left*, 29.64 ± 9.47, max: 44.15, min: 5.63, normality: χ^2^ = 4.0388, *p* = 0.1327)], and in the original target quadrant in reversal probe test [repetitive behavior (Fig 1H *right*, 28.06 ± 9.69, max: 50.76, min: 11.05, normality: χ^2^ = 0.7174, *p* = 0.6986)].

**Fig 2:**
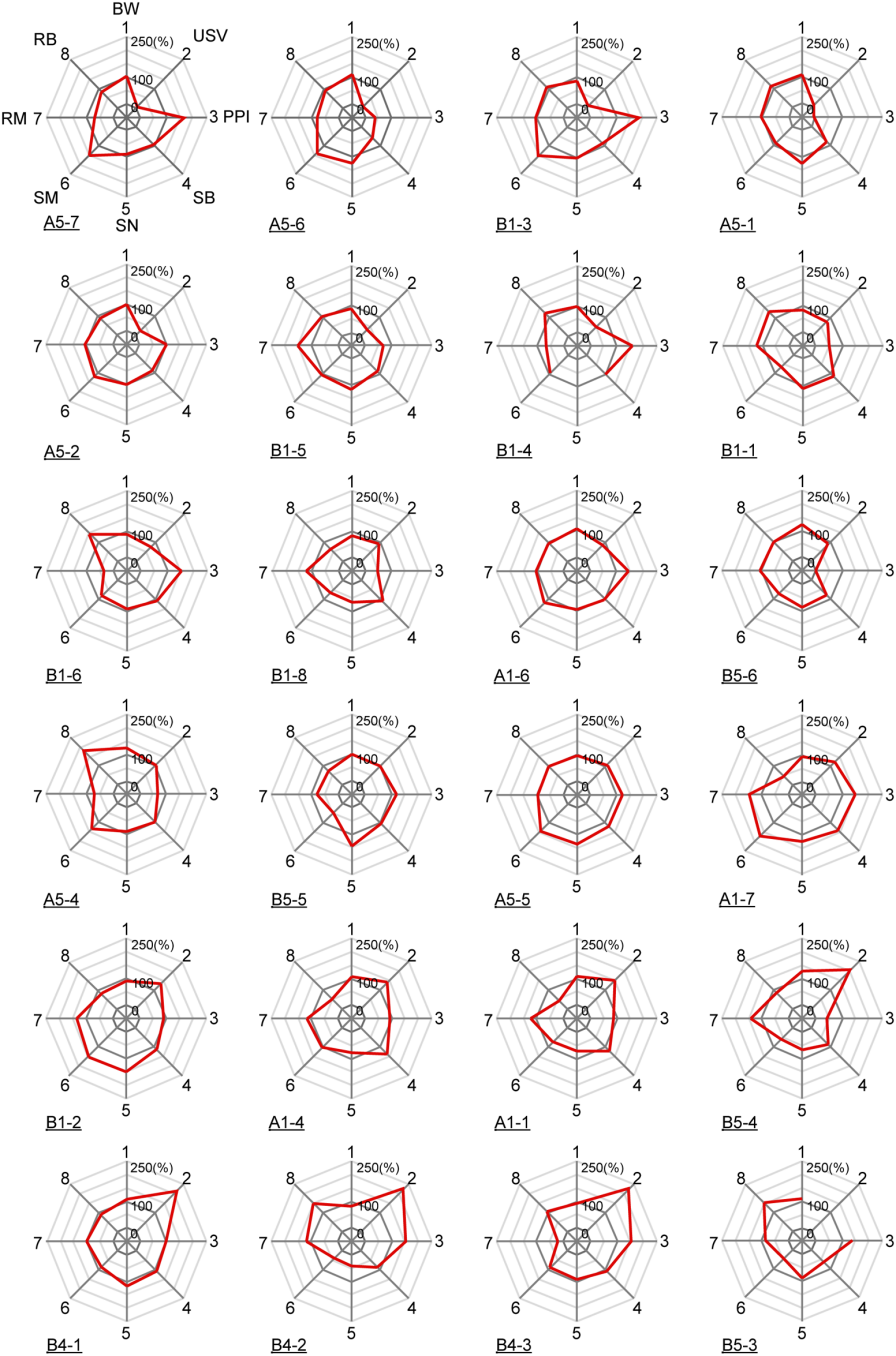
Diversity of behavior scores between individual mice. Body weight and behavioral scores in each mouse from two independent populations (A or B) were represented in the radar chart as relative values. 1: body weight (BW), 2: ultrasonic vocalization (USV), 3: prepulse inhibition (PPI), 4: sociability (SB), 5: social novelty (SN), 6: spatial memory (SM), 7: reversal memory (RM), 8: repetitive behavior (RB).

To understand the individual differences between each mouse, we made radar charts including the body weight at postnatal day 6 and other individual behavior scores of the mice by standardizing to 100% as the average value (Fig 2). The charts clearly showed a diversity individual behavior in inbred mice in our paradigm; there was no mouse that had all scores at the average. This prompted us to examine how each behavior trait was associated with other traits.

### Repetitive behavior was negatively correlated with social behavior in adulthood

**Fig 3:**
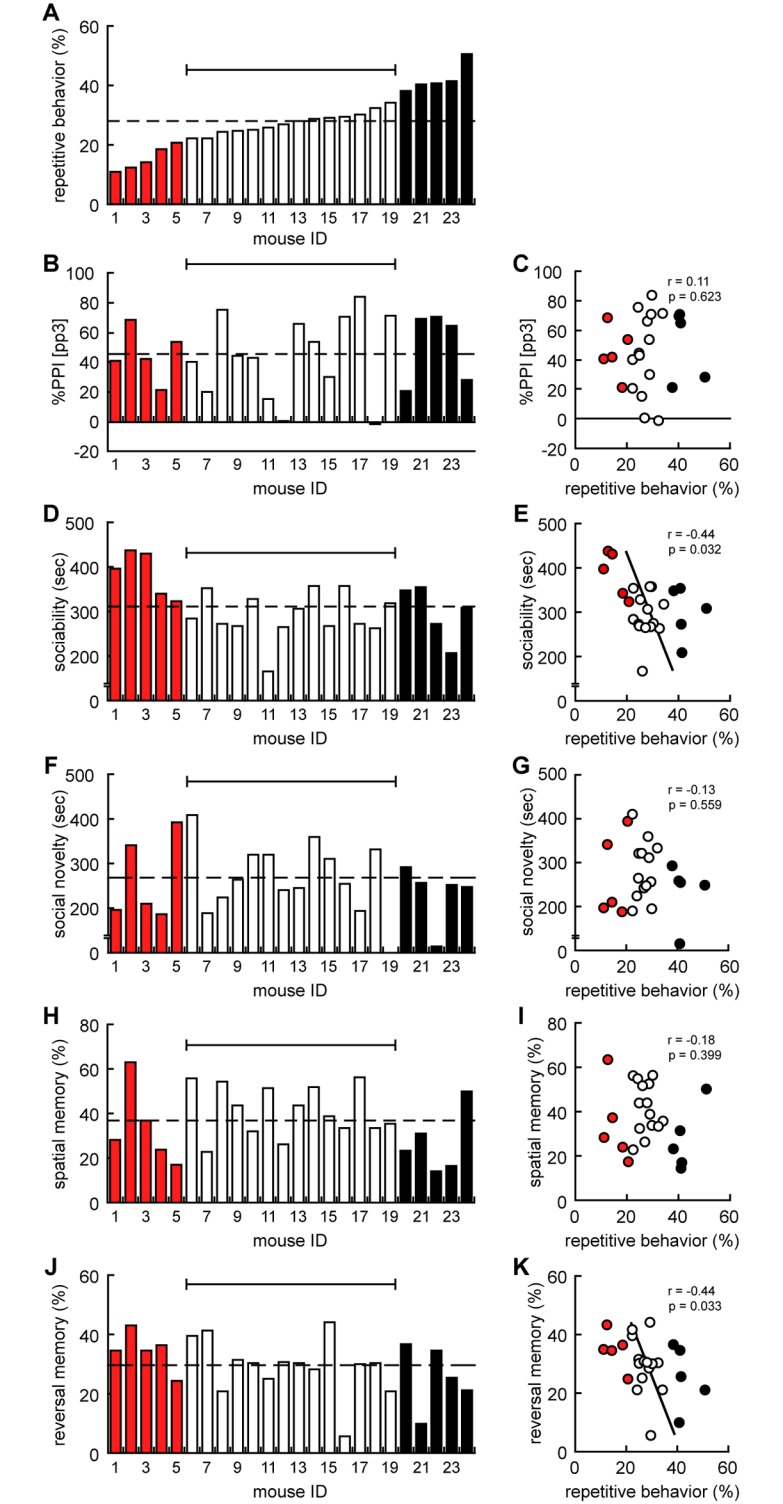
Repetitive behavior was negatively correlated with social behavior in adulthood. (A) Individual data of repetitive behavior (percentage in the original target quadrant in reversal probe test in Morris water maze, MWM) was arranged in the ascending order of powers. (B) Individual data of %PPI (the reduction of the amplitude of startle response by a weaker prestimulus (prepulse)) was arranged in accordance in the following order of individual data in repetitive behavior in MWM. (C) Correlation analysis between repetitive behavior in MWM and %PPI (*r* = 0.11, *t* = 0.501, *p* = 0.623). (D) Individual data of sociability (time spent with stranger mice to the empty cage in 3-chamber social interaction test, 3CSI) was arranged in accordance in the following order of individual data in repetitive behavior in MWM. (E) Correlation analysis between repetitive behavior and sociability in 3CSI (*r* = -0.44, *t* = -2.282, *p* = 0.032). (F) Individual data of social novelty (time spent with stranger mice to familiar mice in 3CSI) was arranged in accordance in the following order of individual data in repetitive behavior in MWM. (G) Correlation analysis between repetitive behavior and social novelty in 3CSI (*r* = -0.13, *t* = -0.593, *p* = 0.559). (H) Individual data of spatial memory (percentage in the original target quadrant in probe test in MWM) was arranged in accordance in the following order of individual data in repetitive behavior in MWM. (I) Correlation analysis between repetitive behavior and spatial memory in MWM (*r* = -0.18, *t* = -0.861, *p* = 0.399). (J) Individual data of reversal memory (percentage in the original target quadrant in reversal probe test in MWM) was arranged in accordance in the following order of individual data in repetitive behavior in MWM. (K) Correlation analysis between repetitive behavior and reversal memory in MWM (*r* = -0.44, *t* = -2.274, *p* = 0.033). Data are presented by histogram or point diagram with a white bar for individual data within (2/3) standard deviation, a red bar for individual data less than (2/3) standard deviation, and a black bar for individual data more than (2/3) standard deviation.

First, we conducted systematic comparisons across behavioral measures among behavior scores obtained in adult (Table 1). We noticed that repetitive behavior was selectively associated with specific behavioral traits, i.e., social behavior and reversal memory. Therefore, individual data of the repetitive behavior scored in MWM were aligned in the ascending order to give an ID for each mouse (Fig 3A). We labeled ‘low repetitive’ (shown in red) and ‘high repetitive’ (shown in black) mice based on the score in MWM, i.e., percentage of time spent in the original target quadrant in the reversal probe was less or higher than two-thirds of the standard deviation, respectively. Then, individual data of %PPI, sociability, social novelty, spatial memory, and reversal memory were shown, according to the mouse ID, based on scores in repetitive behavior (Fig 3B, 3D, 3F, 3H and 3J).

**Table 1:** Correlation coefficients and *p*-value between behavioral results.

	**pup**	**adult**					
	USV	PPI	sociability	social novelty	spatial	reversal	repetitive
USV		0.12(0.601)	**0.45(0.030)**	-0.22(0.329)	**-0.42(0.048)**	0.04(0.866)	-0.11(0.616)
PPI			0.21(0.328)	-0.17(0.439)	0.21(0.340)	-0.34(0.109)	0.11(0.623)
sociability				0.09(0.695)	0.12(0.574)	0.12(0.583)	**-0.44(0.032)**
social novelty					0.34(0.111)	0.03(0.905)	-0.13(0.559)
spatial						0.04(0.864)	-0.18(0.399)
reversal							**-0.44(0.033)**
repetitive							

correlation coefficients (*p*-value)

USV; ultrasonic vocalization, PPI; prepulse inhibition

Warm and cool color represents positive and negative correlation, respectively.

The bold values are statistically significant (*p* < 0.05).

As shown in Fig 3C, there was no correlation between repetitive behavior in MWM and sensorimotor gating scored as %PPI. Also, no statistical correlation was observed between repetitive behavior in MWM with social novelty (Fig 3G) or spatial memory (Fig 3I). In contrast, all ‘low repetitive’ mice showed higher sociability and three of ‘high repetitive’ mice showed less sociability than average (Fig 3D). Moreover, four of the ‘low repetitive’ mice showed higher reversal memory and three of the ‘high repetitive’ mice showed less reversal memory than the average (Fig 3J). Statistical analysis revealed that the score of repetitive behavior in MWM was negatively correlated with the score of sociability in 3CSI (Fig 3E, *r* = -0.44, *p* = 0.032) and with reversal memory in MWM (Fig 3K, *r* = -0.44, *p* = 0.033). Taken together, it is suggested that mice with less repetitive behavior tended to exhibit more sociability and reversal memory in adulthood.

### Higher vocal communication in pups was correlated with higher social behavior but with lower spatial memory in adulthood

**Fig 4:**
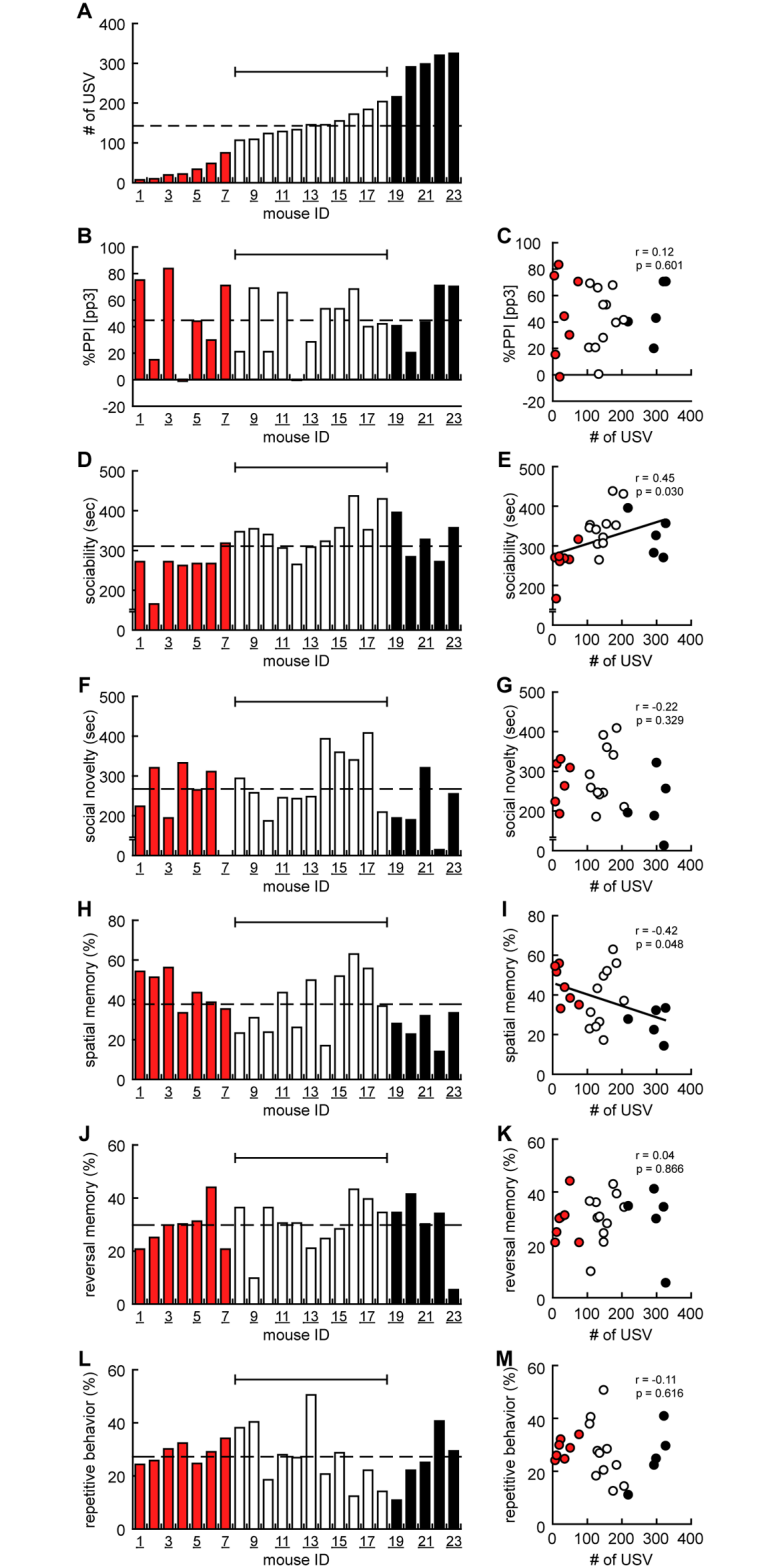
Higher number of USV calls in pups is related with higher social behavior, but lower spatial memory in adulthood. (A) Individual data of ultrasonic vocalization (number of ultrasonic vocalization, USV) was arranged in the ascending order of powers. (B) Individual data of %PPI (the reduction of the amplitude of startle response by a weaker prestimulus (prepulse)) was arranged in accordance with the following orders of USV. (C) Correlation analysis between USV and %PPI (*r* = 0.12, *t* = 0.531, *p* = 0.601). (D) Individual data of sociability (time spent with stranger mice in 3CSI) was arranged in according with the following orders of USV. (E) Correlation analysis between USV and sociability in 3CSI (*r* = 0.45, *t* = 2.334, *p* = 0.030). (F) Individual data of social novelty (time spent with stranger mice to familiar mice in 3CSI) was arranged in according with the following orders of USV. (G) Correlation analysis between USV and social novelty in 3CSI (*r* = -0.22, *t* = -1.001, *p* = 0.329). (H) Individual data of spatial memory (percentage in the original target quadrant in probe test in MWM) was arranged in according with the following orders of USV. (I) Correlation analysis between USV and spatial memory in MWM (*r* = -0.42, *t* = -2.098, *p* = 0.048). (J) Individual data of reversal memory (percentage in the original target quadrant in reversal probe test in MWM) was arranged in according with the following orders of USV. (K) Correlation analysis between USV and reversal memory in MWM (*r* = 0.04, *t* = 0.171, *p* = 0.866). (L) Individual data of repetitive behavior (percentage in the original target quadrant in reversal probe test in MWM) was arranged in according with the following orders of USV. (M) Correlation analysis between USV and repetitive behavior in MWM (*r* = -0.11, *t* = -0.509, *p* = 0.616). Data are presented by histogram or point diagram with a white bar for individual data within (2/3) standard deviation, a red bar for individual data less than (2/3) standard deviation, and a black bar for individual data more than (2/3) standard deviation.

We have also conducted longitudinal correlation analyses of USV scores in pups and behavioral results in adulthood. Individual data were realigned based on USV call data in the ascending order to give an ID for each mouse (Fig 4A). We labeled ‘low-USV (red)’ and ‘high-USV (black)’ pups according to the score in number of USV calls, i.e., less or higher USV calls than two-thirds of the standard deviation, respectively. Then, individual data of %PPI, sociability, social novelty, spatial memory, reversal memory, and repetitive behavior were shown according to the ID given based on the number of USV calls (Fig 4B, 4D, 4F, 4H, 4J and 4L).

No statistical correlations were observed between the score of USV and %PPI (Fig 4C), social novelty (Fig 4G), reversal memory (Fig 4K), or repetitive behavior (Fig 4M). In contrast, there was a statistical correlation between a pup’s USV and sociability in adulthood; the score of USV calls was positively correlated with the score of sociability in 3CSI (Fig 4E, *r* = 0.45, *p* = 0.030). Six of the ‘low-USV’ mice showed less sociability than average, and three of the ‘high-USV’ mice showed higher sociability (Fig 4D). We further compared spatial memory in the ‘low-USV’ and ‘high-USV’ mice. Quite unexpectedly, we found a negative correlation between a pup’s USV and spatial memory in adulthood (Fig 4I, *r* = -0.42, *p* = 0.048). Five of the ‘low-USV’ mice showed spatial memory and all of the ‘high-USV’ mice showed less spatial memory than average (Fig 4H). These findings indicated that mice that emitted the larger number of USV calls in the early postnatal stage tended to exhibit more sociability but less spatial memory in adulthood.

## Discussion

In the present study, we have performed a systematic behavior analysis by individual discrimination of mice. We found diverse behavioral differences between individual inbred C57BL6/J male mice (Fig 2). Therefore, even in genetically-homogeneous mice can show individual differences in their behavior, which suggests the involvement of non-genetic factors in the emergence of “individuality”.

We also noticed intriguing links across the behavioral traits. Firstly, significant correlation was revealed between sociability in 3CSI test and repetitive behavior in MWM test (Table 1). This means that more social mice were less repetitive. In addition to these transverse analyses, we conducted longitudinal analyses. Secondly, we found that the number of USV calls at the early postnatal stage was positively, and quite unexpectedly, negatively correlated with the sociability and spatial memory in adulthood (Table 1). That is, high-USV pups became more social but poorer learners in adult. In this regard, however, we would not make a solid conclusion yet since gender difference and strain specificity were not addressed in this study. Nevertheless, we can speculate that vocal communication in infancy may predict some behavioral characteristics in adulthood.

Several possibilities can explain the correlations between vocal communication in infancy with sociability and spatial memory in adulthood. Firstly, maternal behavior may affect social development of offspring because USV in pups can elicit maternal licking and retrieval behavior [26]. It has also been reported that mice that were more frequently licked and attached by their mother during early postnatal stages were more engaged in social contact with novel and weight-matched conspecific mice than less-licked mice [4]. Intriguingly, less-licked mice have enhanced working memory in the radial arm maze test [27]. Therefore, pups emitting more USV might develop sociability but lack spatial memory in adulthood, possibly through maternal nurturing behavior. Another possibility is that the higher number of USV calls in infancy is based on anxiety that may later induce sociability and impair learning, since maternal separation-induced USV is considered to be an anxiety-related behavior [28]. However, previous literature has reported correlations of excess anxiety induced by prolonged maternal separation with social investigation [29] or cognitive performance [30]. In any case, it may be reasonable to assume that proper pup-mother interactions during the early development might positively development of sociability or negatively affect learning ability in adulthood.

Social impairment and repetitiveness are considered to be core features of autism spectrum disorder (ASD) in the current diagnosis, according to DSM-5. These symptoms, often coexist with other comorbid phenotypes, such as intellectual disability or sensory abnormality, are highly diverse from a mild level to severe level [31]. Some of the autistic symptoms can be modeled in animals [24, 32–35], although individual diversity in each animal has not previously been addressed. In this study, we found that less social individuals exhibited a more repetitive behavioral trait within inbred wild type mice. Similar behavioral variations might be observed in ASD model animals as those shown in wild type mice. ASD patients show remarkable diversity in their traits and some less social individuals sometimes exhibit unique talents, such as genius memory and artistic ability. Therefore, we suggest that more attention should be paid to variability in behavioral studies of animal models.

When and how does individuality emerge? As mentioned above, we found positive and negative correlations in wild type mice between vocal communication in infancy with sociability and learning ability in adulthood, respectively. Thus, USV phenotypes at the early postnatal stage in mice might impact upon sociability and learning ability in adulthood. At this moment, we do not know how such correlations are acquired physiologically or ecologically. Further studies are needed to understand neurodevelopmental mechanisms that operate individual differences such as survey for neuronal activity and neural development of the nucleus associated with behavioral diversity. Nonetheless, our investigations are the first evidence of longitudinal analyses successfully performed in individual inbred mice, which will stimulate new research in neuroscience to understand individual diversity at molecular, cellular, and system levels. To uncover the neural basis underlying differences in vocal communication at the infant stage would contribute to understanding individual diversity in adulthood.
