# Correction: Early postnatal vocalizations predict sociability and spatial memory in C57BL/6J mice: Individual differences in behavioral traits emerge early in development

**DOI:** 10.1371/journal.pone.0191702

**Published:** 2018-01-19

**Authors:** Kaichi Yoshizaki, Kohei Koike, Ryuichi Kimura, Noriko Osumi

The funding information in the Funding section. The correct Funding statement is: This research was supported by JSPS KAKENHI Grant Number 25640002, 15K12764, 16H06530 (NO) and 15K06694 (KY).
